# An Exploration of Novel Bioactives from the Venomous Marine Annelid *Glycera alba*

**DOI:** 10.3390/toxins15110655

**Published:** 2023-11-14

**Authors:** Sónia Campos, Ana P. Rodrigo, Inês Moutinho Cabral, Vera M. Mendes, Bruno Manadas, Mariaelena D’Ambrosio, Pedro M. Costa

**Affiliations:** 1 Associate Laboratory i4HB Institute for Health and Bioeconomy, NOVA School of Science and Technology, NOVA University Lisbon, 2829-516 Caparica, Portugal; sc.campos@campus.fct.unl.pt (S.C.); a.rodrigo@campus.fct.unl.pt (A.P.R.); imf.cabral@campus.fct.unl.pt (I.M.C.); 2 UCIBIO Applied Molecular Biosciences Unit, Department of Life Sciences, NOVA School of Science and Technology, NOVA University of Lisbon, 2829-516 Caparica, Portugal; 3 CNC—Center for Neuroscience and Cell Biology, University of Coimbra, 3060-197 Cantanhede, Portugal; vmendes@cnc.uc.pt (V.M.M.); bmanadas@cnc.uc.pt (B.M.)

**Keywords:** marine invertebrates, Annelida, toxin, venom proteins, toxicity, proteomics

## Abstract

The immense biodiversity of marine invertebrates makes them high-value targets for the prospecting of novel bioactives. The present study investigated proteinaceous toxins secreted by the skin and proboscis of *Glycera alba* (Annelida: Polychaeta), whose congenerics *G. tridactyla* and *G. dibranchiata* are known to be venomous. Proteomics and bioinformatics enabled the detection of bioactive proteins that hold potential for biotechnological applications, including toxins like glycerotoxins (GLTx), which can interfere with neuromuscular calcium channels and therefore have value for the development of painkillers, for instance. We also identified proteins involved in the biosynthesis of toxins. Other proteins of interest include venom and toxin-related bioactives like cysteine-rich venom proteins, many of which are known to interfere with the nervous system. Ex vivo toxicity assays with mussel gills exposed to fractionated protein extracts from the skin and proboscis revealed that fractions potentially containing higher-molecular-mass venom proteins can exert negative effects on invertebrate prey. Histopathology, DNA damage and caspase-3 activity suggest significant cytotoxic effects that can be coadjuvated by permeabilizing enzymes such as venom metalloproteinases M12B. Altogether, these encouraging findings show that venomous annelids are important sources of novel bioactives, albeit illustrating the challenges of surveying organisms whose genomes and metabolisms are poorly understood.

## 1. Introduction

Venomous secretions are complex mixtures of bioactives, from toxins and permeabilizing enzymes to salts and peptides, that are synthesized in specialized gland cells and conveyed to the target organisms via a wound, thereby disrupting normal physiological processes [1,2]. Due to the long-term coevolution between predator and prey, proteinaceous toxins in venoms may gain increased potency and high specificity, and, in general, we may expect a span of toxins adapted to interact with a considerable range of molecular targets such as cell receptors and membrane ion channels to maximize the full potential of venoms [3]. Not surprisingly, the exploration of biological toxins may thus become an alternative to the expensive and time-consuming process that is the design of synthetic pharmaceuticals (reviewed elsewhere [4,5]).

Being the largest and most ancient habitat on Earth, the marine environment bears an immense biodiversity that correlates with an almost endless span of novel bioproducts resulting from selective pressure and adaptation to its diversified ecosystems. As such, the ocean is an auspicious target for bioprospecting for new bioactive products, with an emphasis on novel marine toxins with potential applications in several biotechnological domains including pharmaceuticals [6,7]. For example, “Hemiasterlin” is a tripeptide with cytotoxic properties isolated from the marine sponge *Hemiasterella minor*. This toxin blocks cell division during the M phase of mitosis by targeting tubulin, hindering the formation of the spindle; it has also advanced to Phase II clinical trials as an antitumor natural product [8]. As another example, some nemertines secrete toxins capable of paralyzing their prey and deterring predators by acting as agonists of the central nervous system’s nicotinic receptors (acetylcholine receptors) and cardiac ganglion. These compounds were investigated to improve cognitive impairment in patients with Alzheimer’s disease, since these patients have been shown to have a deficiency in nicotinic synaptic transmission [9,10]. Another example is *Arenicola marina*, a tubicolous marine annelid that produces two peptides, arenicin-1 and arenicin-2, with activity against both Gram-positive and Gram-negative bacteria as well as fungi [11,12]. Furthermore, from an ω-conotoxin of the gastropod mollusk *Conus magus*, the nonopioid analgesic “Ziconotide” was developed, whose action is considered as strong as morphine [8,13]. It must be noticed, however, that despite the large number of natural toxins currently being added to libraries, research has been focused on a relatively small number of marine species, with an emphasis on tropical organisms, particularly considering the ocean’s immense invertebrate biodiversity and the unsuspected richness of temperate waters. In fact, a broad range of marine invertebrates such as annelids, mollusks and cnidarians, regardless of latitude, secrete toxins and other bioactives for defense and predation. From this perspective, these organisms are especially interesting due to their vast molecular diversity driven by unique adaptive traits, a result of adaptation to a broad range of habitats, from beaches to abyssal plains [12,14].

Putative marine bioproducts have already been pinpointed from marine Annelida, but research so far likely only offers a glimpse of their true potential [4]. This large polyphyletic (and now considered deprecated) class of annelids (mostly marine) encompasses the gross of the Annelida diversity [12]. They are found from intertidal areas to deep-sea vents and, therefore, can be very morphologically distinct depending on ecology, despite following the same basic body plan [15]. Novel bioproducts from marine annelids are just beginning to be unraveled, and natural products from these animals effectively lack commercial applications despite indications of their promising potential. This includes fluorescent probes retrieved from the mucus of *Eulalia* sp. [16] and the photoprotein-based bioluminescence of *Odontosyllis phosphorea* [17]. We may add potential painkillers and anesthetics that may be developed from neurotoxins like those described in some *Glycera* species [18] that are similar to Prialt, an approved drug developed from the venom of the cone snail *Conus* (see Rodrigo and Costa [4] for further examples on potential biotechnological applications of polychaetes).

Glycerids, also known as bloodworms, are carnivorous marine annelids that typically feed on other annelids and small crustaceans, injecting a venom mixture through an eversible proboscis that should cause cardiac arrest, progressive paralysis, convulsions and, eventually death in their prey [18,19,20]. The proteinaceous toxins identified from *Glycera tridactyla*, *G. fallax* and *G. dibranchiata* venom glands can be placed into five categories: pore-forming, neurotoxins, protease inhibitors, CAP domain toxins and permeabilizers [18]. Particularly, the venom of *G. tridactyla* contains glycerotoxin (GLTx), a high-molecular-mass neurotoxic glycoprotein (150 kDa, potentially forming 300–320 kDa dimers) that induces Ca^2+^ influx at resting potential, which reversibly increases spontaneous neurotransmitter release [21,22,23]. Additionally, the same species produces toxins considered as homologs of proteins known only from scorpaeniform fishes and monotremes, namely, SNTX-like toxins [18]. Common in the Portuguese intertidal, the bloodworm *Glycera alba* (O.F. Müller, 1776) is a predator that, like other glycerids, uses its eversible proboscis to ensnare its prey. Novel studies provided strong evidence for the secretion of venoms as well, including the presence of glands connected to four chitinous jaws [24,25]. The present work contributes to the study of the venom of glycerid worms by addressing the composition and bioactivity of proteinaceous toxins and other bioactives secreted by *G. alba*. Specifically, we aim to (1) investigate the presence of glycerotoxin forms in the worms’ secreted proteome as well as other bioactives of interest, and (2) investigate the bioactivity and toxicity of the crude venom and attempt to relate it to its putative composition.

## 2. Results

### 2.1. Comparative Proteomics between Proboscis and Skin

#### 2.1.1. Gel Electrophoresis of Crude and Fractionated Protein Extracts

**Figure 1:**
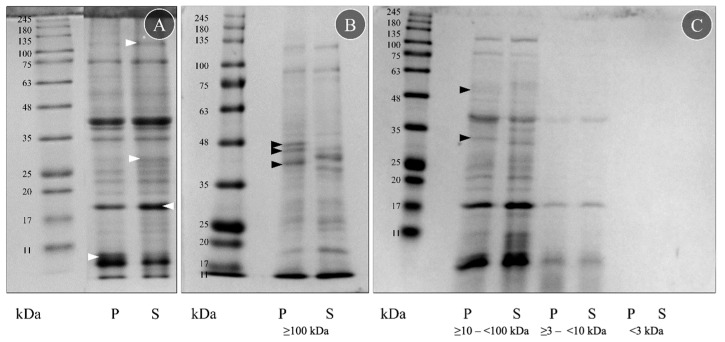
Protein signature of crude and fractionated extracts from *G. alba* proboscis (P) and skin (S). (**A**) Protein samples were diluted to the same protein concentration (2.5 mg mL^−1^) in Milli-Q-grade ultrapure water before SDS-PAGE. For comparison, the more intense bands are marked with a white arrowhead. (**B**) Fraction obtained with ≥100 kDa ultrafiltration columns. (**C**) Fractions obtained using ≥10–<100 kDa, ≥3–<10 kDa and <3 kDa filters. Fractionated protein samples were diluted to the same amount of protein (3 µg) in PBS before the SDS-PAGE. Bands found only on proboscis are marked with a black arrowhead. The molecular standard used was the NZYColour Protein Marker II (Nzytech) in the range of 11–245 kDa.

Four crude protein extracts were obtained from the proboscis (P) and skin (S) crude extracts using ultrafiltration columns targeting different molecular-mass biomolecules. The proteins and peptides in the extracts were then separated using SDS-PAGE (Figure 1). Separation yielded scant differences between the two organs, displaying almost identical proteomes (Figure 1A). Apart from some differences in intensity, especially in the skin extract (white arrowheads, Figure 1A), due to different protein quantities, both proteomes displayed the same protein bands. The reduced smearing indicates that the extraction and storage process did not significantly contribute to protein degradation.

The SDS-PAGE analyses of the four fractions of the crude extracts from the proboscis and skin disclosed important differences between the organs within the fractions obtained with the highest-molecular-mass filter, i.e., ≥100 kDa (Figure 1B), and by using the ≥10–<100 kDa pair of filters (Figure 1C). In these two fractions, five bands were visible only in the proboscis (P): three between 35 and 48 kDa (Figure 1B, black arrowheads), one from 25 to 35 kDa and one from 48 to 63 kDa (Figure 1C, black arrowheads). However, the remaining fractions (obtained with ≥3–<10 kDa and <3 kDa filters) did not display clear differences (Figure 1C). Despite all protein samples being diluted to the same amount of protein, 3 μg, the ≥3–<10 kDa filter fraction was less intense than the two higher-molecular-mass ones, and the <3 kDa filter did not present a clear signal. The fractions obtained with the ≥10–<100 kDa and ≥3–<10 kDa filters had a very similar protein signature to the crude extracts, contrasting with the ≥100 kDa fraction that presented more distinct differences between the organs. The fraction that yielded the most crucial differences between the two organs, the one produced with the higher-molecular-mass filter (≥100 kDa), was then tested for toxicity on mussel gills.

The fractioned proteins were compared with the described molecular mass for toxins known from other *Glycera* spp. (Table S1). According to von Reumont et al. [18], *G. dibranchiata*, *G. fallax* and *G. tridactyla* toxins are classified into five categories: pore-forming toxins, neurotoxins, protease inhibitors, CAP domain toxins and other enzymes (C-type lectin, chitinase, hyaluronidase, phospholipases, peptidase S1, peptidase S10 and metalloproteinase M12). The SDS-PAGE gels suggested seven possible peptide matches in *G. alba* based on molecular mass: serpins, chitinases, peptidase S1 and S10, hyaluronidases, lipocalins and metalloproteinase M12.

#### 2.1.2. Proteomics

The analysis of proteins and peptides by LC-MS/MS yielded a total of 165 putative proteins with a positive identification against the customized database that included toxin- and venom-related proteins (Table S2). Of these, 63 were only identified in samples from the proboscis, 45 only in the skin and 57 common to both organs (Figure S1). Glycerotoxin (GLTx) was present among several toxin candidates. In the proboscis, two potential GLTx homologs with a more robust coverage were found, together with a higher number of matching peptides in both replicates (Table 1). In skin extracts, only one peptide from one of the replicates could be matched against GLTx. Contrasting proteomic against transcriptomic data for *G. alba* retrieved a total of eight peptides matched against three predicted open reading frames (ORFs) that were overexpressed in the proboscis relative to the skin with significant homology matching to GLTx (see Moutinho Cabral et al. [26]). Despite the high percentage of identity (90% to 100%), the *e*-value ranged between 5.75 × 10^−8^ and 8.46 × 10^−6^. On the other hand, the homology search of all annotated peptides against the three GLTx sequences from UniprotKb confirmed previous matches against the entire database, yielding a total of 42 hits with a percentage of identity of 100% and an *e*-value ranging between 2.18 × 10^−15^ and 4.23 × 10^−6^.

**Figure 2:**
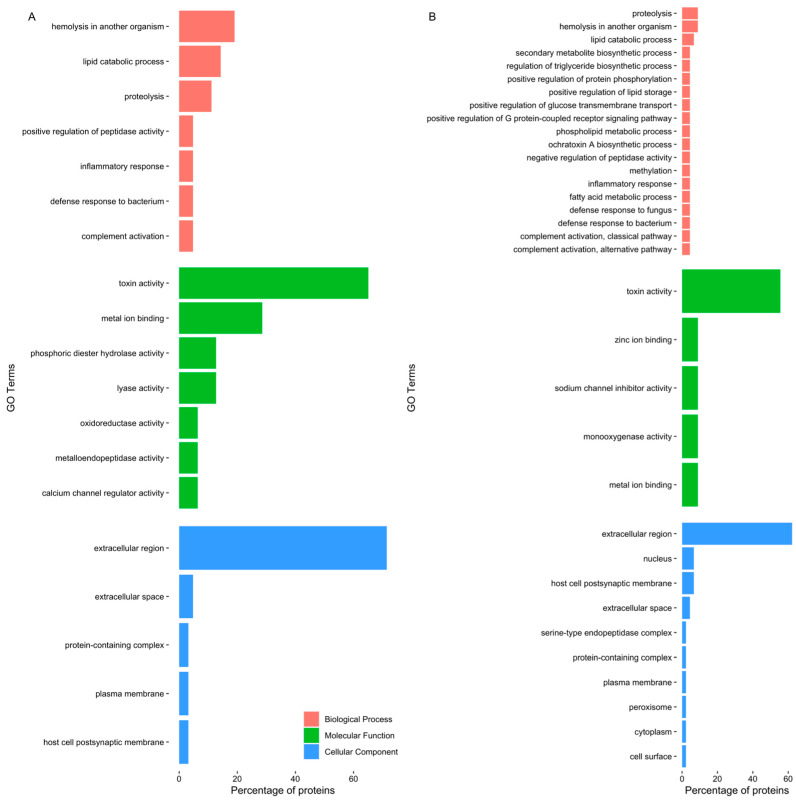
Top five GO terms of proteins identified in *G. alba*’s crude protein extracts. The horizontal bar chart shows the percentage of the proteins exclusively identified, related to each GO term, in (**A**) the proboscis, and (**B**) the skin. Note that the same protein can bear multiple GO terms.

Gene ontology analysis disclosed a pattern of proteins related to toxin and other venom components (Figure 2). Even though the most frequent GO terms were “toxin activity” and “extracellular region”, differences were identified between the two different extracts. The GO term “calcium channel regulator activity” was highlighted in the proboscis, as it was related to GLTx and other toxins such as cysteine-rich venom proteins as well as omega-hexatoxin. Furthermore, “phosphoric diester hydrolase activity” and ”metalloendopeptidase activity” were also noteworthy, as they related to dermonecrotic toxins (sphingomyelinase D) and venom metalloproteinases M12B (Table S2). When only considering the proteins in common between the proteomes of the two organs, the proteins identified were related to biological processes, such as biosynthesis of toxins, immune response and proteolysis (Figure S2). Moreover, when considering all proteins found in the proboscis (Figure S3A), there was a link with biological processes such as “hemolysis in another organism”, “proteolysis” and “inflammatory response”, while in the skin (Figure S3B), proteins were chiefly related to “inflammatory response”, “proteolysis” and “complement activation, classical pathway”.

### 2.2. Toxicity Testing

#### 2.2.1. Histopathological Analysis of Mussel Gills

**Figure 3:**
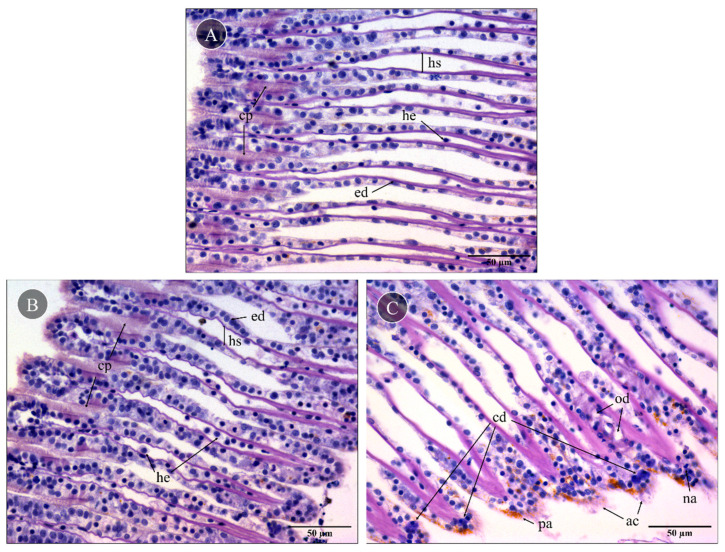
Representative histological gill sections of *Mytilus* sp. Gills exposed ex vivo to extracts from the proboscis and skin of *G. alba* for 10 min (1 mg total protein mL^−1^), stained with hematoxylin and eosin (H&E). (**A**) Control gill, exposed to PBS. Note the presence of intact ciliary plates (cp), the hemolymphatic sinus (hs), endothelial cells (ed) and a few hemocytes (he) in vessels. (**B**) Gill exposed to skin extract (1 mg mL^−1^ total protein in PBS). No visible alterations compared to the basal levels. (**C**) Gills exposed to proboscis extract (1 mg mL^−1^), revealing the presence of edema (od), early stages of cell death (cd), hemocytes with nuclear abnormalities (na), abrasion of cilia (ac) and pigment accumulation (pa). Scale bars: 50 µm.

Mussel gills exposed to PBS (control) showed no significant lesions in the gill epithelia and hemolymph vessels (Figure 3A). Control gills had a well-preserved morphology, with two lamellar structures composed of filaments bound by ciliary plates (cp), hemolymphatic sinuses and endothelial cells. Also, the delicate ciliary plates were intact, and only a few hemocytes could be found in the sinuses, which excluded significant inflammatory response. Compared to the control, gills exposed to skin extracts for 10 min (1 mg total protein mL^−1^) did not have visible alterations (Figure 3B). On the other hand, gills exposed to the proboscis extract with the same protein concentration (Figure 3C) for 10 min as well yielded moderate but persistent levels of histopathological alterations to the epithelia. Specifically, focal edematous fluid retention between cells was evident, as was the presence of hemocytes with nuclear abnormalities, mild abrasion of cilia and brown pigment (lipofuscin-like) accumulation (Figure 3C). Additionally, cells revealing a compact nucleus with denser blueish cytoplasm indicated early stages of cell death. The lack of a strong inflammatory response, which should imply more noticeable hemocyte migration, plus intact cell membranes suggested apoptosis instead of necrosis.

#### 2.2.2. DNA Damage in Mussel Gills

**Figure 4:**
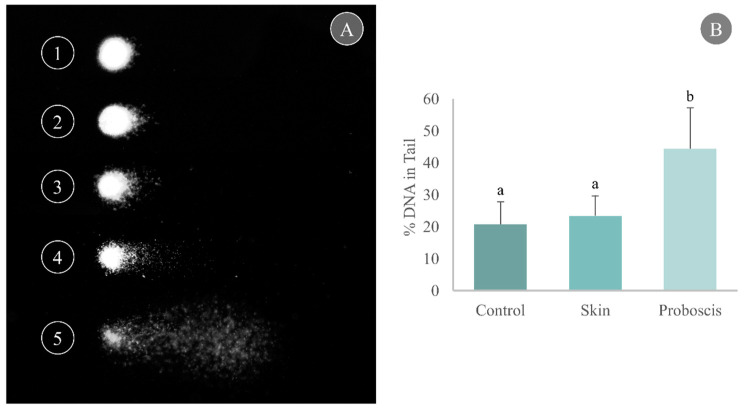
Determination of DNA damage through the comet assay in cells from mussel gills exposed ex vivo to *G. alba*’s crude protein extracts for 10 min (1 mg total protein mL^−1^). (**A**) Representative nucleoids of comet classes based on % DNA in tail: (1) 0–20%, (2) 20–40%, (3) 40–60%, (4) 60–80%, (5) 80–100%. (**B**) Comet assay results expressed as % DNA in tail in the gills of mussels exposed ex vivo (1 mg mL^−1^ crude protein in PBS) to proboscis or skin extracts, plus controls (gills treated with PBS only). Results are expressed as means + standard deviation. Different letters indicate significant differences (Dunn’s test, *p* < 0.05).

Cell harvesting from gills yielded good-quality nucleoids for the comet assay with a unimodal distribution per slide, thus indicating reduced influence of necrotic and apoptotic cells (Figure 4A). Gill cells exposed to the skin crude protein extract for 10 min attained a maximum mean of 23.4% DNA in the tail, marginally higher than the control (20.7% DNA in the tail). On the other hand, exposure to the proboscis extract for 10 min caused a significant increase in the percentage of damaged DNA in mussel gills, reaching a maximum mean of 44.3% DNA in tail (Dunn’s test, *p <* 0.05, Figure 4B).

#### 2.2.3. Caspase-3 Activity as an Indicator of Apoptosis

**Figure 5:**
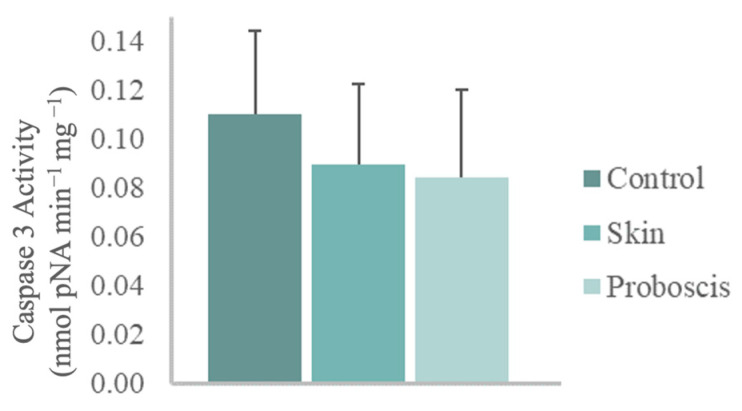
Caspase-3 activity in mussel gills exposed ex vivo (10 min) to *G. alba*’s crude protein extracts from proboscis and skin (1 mg total protein mL^−1^). Normalized caspase-3 activity in mussel gills exposed to proboscis and skin extracts and control (gills treated with PBS). The results represent the biological replicates’ mean normalized activity + standard deviation, estimated from the p-nitroaniline (pNA) absorbance at 405 nm.

The 10 min ex vivo exposure to the crude protein extract (1 mg total protein mL^−1^) from *G. alba*’s proboscis yielded a mean caspase-3 activity of 0.085 nmol pNA min^−1^ mg^−1^ in mussel gill cells. In turn, gills exposed to the skin extract for the same duration and total protein concentration attained a mean caspase-3 activity of 0.09 nmol pNA min^−1^ mg^−1^. However, no significant differences relative to control gills (mean caspase-3 activity of 0.11 nmol pNA min^−1^ mg^−1^) were found (Figure 5).

## 3. Discussion

The current study demonstrated that *G. alba* secretes proteinaceous bioactives that are consistent with biological toxins similar to other known congenerics. This outcome is supported by molecular characterization of crude protein extracts from the worm’s proboscis and from toxicity testing, even though testing needs to be further refined to clearly isolate the effects from the crude venom and isolated toxins. Altogether, the toxicity assays indicate that *G. alba*, like other congeners, likely secretes high-molecular-mass toxins and accompanying enzymes that can exert negative effects on their prey, making them an important target for marine-based drug discovery due to their bioactivity and potential selectivity.

The proteome profiles of the proboscis and skin organs yielded many similarities. However, a cautionary interpretation of data is needed, especially because both the proboscis and skin of *G. alba* are highly muscularized organs [19,27], which could mean that organ-specific proteins will likely be similarly overshadowed by myocyte proteomes. Still, annotation against a customized database that essentially allocated toxins and other venom components should avoid, at least in part, overshadowing of toxins and other venom components by abundant structural proteins, especially in low-genomic-annotated organisms. Indeed, the SDS-PAGE of crude extracts revealed similar band profiling, which accords with the presence of abundant muscle proteins, even though a previous comparison between both organs based on morphoanatomy and histochemistry disclosed the presence of specialized glandular tissue in the proboscis, suggesting that this is, in fact, the main venom-secreting organ [24]. As such, differences between secreted proteomes were expected a priori and would be likely associated with toxins and permeabilizing agents, among other bioactives. It must be noted, however, that the current study is a primary approach to an unknown proteome. Fractioning and increased sample size for quantitative analyses are aspects to be improved in future work.

Regardless of constraints, the methodology enabled the identification of proteinaceous toxins, especially in the proboscis, with emphasis on GLTx, a toxin that has been deemed of biotechnological interest. With more than 60 *G. alba* peptides matched against glycerotoxins (GLTx) from *G. tridactyla*, 25 of which were shared between replicates (out of a total of 29), there is a probable identification of a form of neurotoxin or its precursors in *G. alba*’s proteome. As previously mentioned, GLTx upregulates the activity of calcium channels, which increases spontaneous neurotransmitter releases [21], and this has possible applications in drug development for chronic pain. Furthermore, our study indicates that GLTx is produced and secreted by the proboscis, and it might also have multiple forms, which is corroborated by the work of Richter et al. [22], who located GLTx expression within the pharyngeal lobes. It also emphasizes a link between the transcripts coding for GLTx identified in the proboscis of *G. alba* (refer to Moutinho Cabral et al. [26]) and the peptides matching to the specific toxins of *Glycera* in this study, thus, validating the results.

Homology matching also identified toxins and other venom components of interest in both organs, as well as proteins involved in the biosynthesis of toxins. Nonribosomal peptide synthetase TES, involved in the production of tentoxin in a fungus, is highlighted as we found matching peptides across all replicates in the proboscis and the skin (four to nine peptides). Tentoxin is a phytotoxin that might induce chlorosis in specific plants [28,29], and therefore, the identification of a homolog in *G. alba* that is involved in its biosynthesis revealed that this marine species might produce substances with potential for biotechnological applications, mainly as an herbicide. On the other hand, most of the proteins identified exclusively in one organ, or both, each had a low number of peptides matching against it (less than seven), and consequently, caution is mandatory during the proteomic analysis. For instance, only one peptide was identified in the skin as a homolog to GLTx. Thus, this raises the question of whether the peptide is a toxin precursor or a result of residual expression. Also noteworthy is the matching against a few venom- and toxin-related proteins highlighted by the GO analysis. These toxins might also have biotechnological applications, namely, cysteine-rich venom proteins that might be used to manage and treat diseases of the nervous system (see Table S2). These proteins have already been described in distinct venoms and are neurotoxins as they might block calcium and potassium channels, thus preventing muscle contraction [30,31]. Another neurotoxin identified that might have biotechnological applications is omega-hexatoxin, since it may inhibit voltage-gated calcium channels in insects [32,33]. Furthermore, multiple venom metalloproteinases M12B were identified. that were already reported in different venoms from marine annelids [34,35], snakes and spiders, where they described to be prohemorrhagic [36,37]. These proteins, and potentially other proteinases may permeabilize the tissue to facilitate toxin infiltration. Another interesting result was the identification of multiple dermonecrotic toxins (sphingomyelinase D) through a single peptide. These toxins are described as provoking the formation of edemas, inflammatory responses, hemolysis and dermonecrosis [38]. Despite the reduced quality of matching, these findings reiterate the potential of *Glycera*’s proteome. Altogether, this proteomic approach and the study of the *G. alba* transcriptome [26] confirmed that the proboscis might secrete specific toxins, such as GLTx, along with diffusing and permeabilizing agents to overpower its prey. This also suggests protein synthesis in the proboscis, as expected from an organ adapted for venom secretion.

Protein separation of fractionated extracts revealed further evidence for venom secretion. The two higher-molecular-mass fractions obtained from both proboscis and skin (≥100 kDa and 10–100 kDa) showed differences in the ranges of 48–63, 35–48 and 25–35 kDa when compared to the fractions with lower molecular mass. Furthermore, such proteins may contain CAP domains and bear neurotoxic properties, such as cysteine-rich secretory proteins (CRISPs), characterized by multiple thiols and seemingly abundant in *G. alba* venom glands, as suggested by Gonçalves and Costa [24]. It must be noted that cysteine-rich proteins are common in animal venoms and poisons from snakes to gastropods (e.g., D’Ambrosio et al. [39]; Heyborne and Mackessy [40]), being low-molecular-mass proteins with high reactivity and nucleophilicity due to sulfhydryl groups.

Gene ontology analysis was generally accordant with the toxicity assays and the preliminary assays reported by D’Ambrosio et al. [25], as the extract from the proboscis of *G. alba* caused DNA damage and cellular apoptosis in mollusk gills. These findings indicated that this extract bears a higher cytotoxic effect on gill cells compared to skin proteinaceous secretions. Additionally, histological analysis of gills cells exposed to proboscis extract (Figure 3) evidenced edematous fluid retention as a consequence of hindered osmotic equilibrium, nuclear abnormalities in hemocytes and brown pigment (lipofuscin) accumulation, which may be associated with tissue clearance by defense cells (see, for instance, Costa [41]). Despite the evidence for apoptosis from histopathology and DNA fragmentation through the comet assay, the activity of caspase-3 was unchanged between experimental conditions. Since caspase-3 is the most studied mammalian effector caspase involved in the intrinsic pathway [42], the lack of significant differences could be explained by the triggering of the extrinsic pathway. Indeed, it may be argued that toxinlike proteins likely act on surface receptors of target cells, therefore activating apoptosis by the extrinsic pathway. This pathway acts by activating the death of cellular membrane receptors of the TNF family, whereas the intrinsic pathway involves the action of mitochondria following cytotoxic insult such as excessive DNA damage. Such effects on programmed cell death have already been described, in fact, in human cancer cells exposed to proteinaceous secretions from a fellow Phyllodocida [43]. It must be noted that as caspase-3 is an effector caspase involved in the intrinsic pathway, its activity would not have increased upon exposure, leading to the same activity for all organs [42]. Still, we must also consider that the kit used was designed for mammals and not bivalves. As such, the commercially supplied substrate may not be compatible with a molluscan caspase-3. Indeed, previous research revealed that the bivalve apoptotic and inflammatory pathways are different from the ones in vertebrates and suggested that there might not exist a direct homolog to certain caspases, including executioner caspases that have unique motifs in the prodomains [44].

The tested protein extracts were a mixture of all types of proteins found in each respective structure; they were not composed of purified toxins, containing structural proteins from muscle tissue and epithelium as well as other peptides. One consequence is that the quantities of each component in every extract can vary. Nevertheless, variations, in this case, seem to be negligible, as the usage of biological replicates gave similar results. Another important aspect to note is that *G. alba*’s venom causes cardiac arrest, progressive paralysis and convulsions as result of effects on the nervous system, whereas gills are mainly composed of epithelial tissue. In addition, *G. alba*’s typical method of toxin delivery is injection, not diffusion. Altogether, the observed negative effects may be subdued when compared to the *Glycera*’s natural venom injection.

In conclusion, cytotoxic effects can indicate toxin specificity and eventually even mode of action, which in turn leads to high biotechnological potential for drug discovery. A combination of proteomics with toxicity testing validated the evidence of toxins and the classification of *G. alba* as a venomous animal like its congenerics. Altogether, these findings emphasize the relevance of marine annelids in bioprospecting for novel proteinaceous bioactives for potential biomedical applications. Nonetheless, it is clear that the disclosure of the biotechnological potential of toxins and crude venoms from these organisms needs a better understanding of the role of each individual toxin and accompanying protein in the mixture and a refinement of toxicity testing towards specific endpoints that may heavily depend on biological target and time or dose responsiveness.

## 4. Materials and Methods

### 4.1. Animal Collection

Adult *Glycera alba* (30–80 mm length and 100–300 mg total mass) were hand-collected during low tide from the sandy–muddy intertidal flat in the bay of Seixal, Portugal (38°38′40.7″ N 9°06′07.8″ W). Specimens were transported alive to the laboratory and kept in a mesocosm environment, an aquarium with approximately 7 cm of sand depth and 7 L of artificial saltwater in a closed-circulation system with controlled temperature and photoperiod, with continuous aeration and water recirculation. The period of acclimatization lasted ca. one week before animal processing. Salinity, temperature and photoperiod were restricted to 30, 17 ± 1 °C and 16:8 h light:dark cycle, respectively. For the toxicological assay, *Mytilus* sp. (*M. edulis*/*galloprovicialis* complex), 35–45 mm length and 8–10 g total mass, were hand-collected during low tide from a rocky intertidal area at Costa da Caparica, western Portugal (38°38′28″ N 9°14′18.9″ W). Mussels were acclimated to lab conditions (one–two weeks) in the closed-circulation system described above, in separate aquaria from the worms.

### 4.2. Protein Extraction

Total protein was extracted from the proboscis (where the venom apparatus is in *Glycera* spp.) and skin of eighty-three worms that were immobilized by hypotonic shock and then microdissected, ensuring that internal organs remained unscathed. Skin samples, used as a reference organ, included the epidermis and the underlying musculature. The proboscis included the venom glands, ducts and pharyngeal lobes [22], which are too minute and delicate to be excised individually without significant or total loss of proteinaceous secretions for analyses. The target organs were homogenized in cold separately with a pestle in cold buffer (200 mM DTT, 0.05 M Tris-HCl, pH 7.0) complemented with 1% *v*/*v* Protease Inhibitor Cocktail (Sigma-Aldrich, St. Louis, MO, USA). Samples were then centrifuged for 5 min, ≈12,000× *g*, at 4 °C. The supernatants were pooled, with each pool containing extracts from seven animals, and stored at −80 °C until further processing. Protein concentration was determined using a NanoDrop 2000 spectrophotometer (Thermo Fisher Scientific, Waltham, MA, USA).

#### 4.2.1. Protein Electrophoresis

The discontinuous gel system [45] was used for the one-dimensional separation of proteins in crude extracts under denaturing conditions by sodium dodecyl sulphate-polyacrylamide gel electrophoresis (SDS-PAGE), as detailed by Hames [46]. Resolving and stacking gels contained 12% (*v*/*v*) and 6% (*v*/*v*) acrylamide, respectively. All lanes were loaded with 25 μg of protein and diluted using the previously described buffer. The running buffer consisted of 25 mM Tris, 192 mM glycine and 3.5 mM SDS. The molecular standard used was NZYColour Protein Marker II (Nzytech, Lisboa, Portugal), range 11–245 kDa. Gels were stained overnight with Coomassie Brilliant Blue R-250 and washed with Milli-Q-grade water.

#### 4.2.2. Proteomics

Crude protein extracts of proboscis and skin underwent shotgun proteomics through liquid chromatography coupled with tandem mass spectrometry (LC-MS/MS). Protein for analysis was retrieved from SDS-PAGE gels to remove salts and other substances. The resolving and stacking gels contained 12% (*v*/*v*) and 4% (*v*/*v*) acrylamide, respectively. All wells were loaded with 200 μg of protein from two different replicates for each organ. The SDS sample buffer consisted of 70% (*v*/*v*) Tris-HCl, pH 6.8, 30% (*v*/*v*) glycerol, 10% (*m*/*v*) SDS, 0.6 M DTT and 0.012% (*m*/*v*) bromophenol blue. The gel was stained overnight with Coomassie Brilliant Blue R-250 and washed with Milli-Q water. The gel bands were excised into tubes with sodium azide (0.05%) and stored at 4 °C until destaining and digestion using porcine trypsin. The analysis was performed on a NanoLC™ 425 System (Sciex, Framingham, MA, USA) coupled to a TripleTOF 6600 mass spectrometer (Sciex). The peptides were separated in a Triart C18 Capillary Column 1/32” (12 nm, S-3 µm, 150 × 0.3 mm, YMC, Kyoto, Japan) through chromatography by micro-LC at 50 °C. The parameters were set as follows: flow rate (5 μL min^−1^), mobile phases A (0.1% *v*/*v* formic acid, 5% *v*/*v* DMSO in water) and B (0.1% *v*/*v* formic acid, 5% *v*/*v* DMSO in acetonitrile). The ESI DuoSpray ionization source was conducted in the positive mode set to an ion spray voltage of 5500 V, 25 psi for both nebulizer gas 1 (GS1) and curtain gas (CUR). The rolling collision had a collision energy spread of 5. ProteinPilot 5.0.1 (Sciex, Toronto, ON, Canada) was used for mass fingerprinting with the following parameters: digestion by trypsin, cysteine alkylation by acrylamide and gel-based ID as a special factor. The resulting amino acid sequences were contrasted against a customized database using Blast [47,48]. Briefly, this database was built from a subset of UniProtKB (release 2022_01) [49] that contained manually annotated toxin- and venom-related proteins from eucaryotes (excluding human) that were not localized in the membrane or cytoskeleton, plus unreviewed proteins from Annelida with similar filter criteria. The accuracy of identification was determined through the number of matching peptides per protein and the 95% CI percentage of coverage.

#### 4.2.3. Protein Fractioning

Protein fractions were obtained by subjecting the crude protein extracts from proboscis and skin to ultrafiltration using molecular mass (MM)-ranked filters, namely, 100 kDa, 10 kDa and 3 kDa Amicon spin column filters (Merck KGaA, Darmstadt, Germany), following manufacturer instructions using a refrigerated centrifuge. The three filters were used in series to obtain four different fractions, henceforth termed ≥100 kDa; ≥10–<100 kDa; ≥3–<10 kDa and <3 kDa. The buffer (200 mM DTT, 0.05 M Tris-HCl, pH 7.0) complemented with 1% *v*/*v* Protease Inhibitor Cocktail (Sigma-Aldrich) was replaced with Dulbecco’s phosphate-buffered saline (PBS), pH 7.4, a physiological compatible media, through the column filters and was then used as a vehicle. The proteinaceous nature of the fractions was assessed by SDS-PAGE as previously described. The resolving and stacking gels contained 9% (*v*/*v*) and 6% (*v*/*v*) acrylamide, respectively, for the ≥100 kDa fraction and 12% (*v*/*v*) and 6% (*v*/*v*) acrylamide, respectively, for the remaining fractions. Wells were loaded with extracts diluted to the same amount of protein (3 μg) in PBS. The protein concentration of each fraction was determined using a NanoDrop 2000 spectrophotometer (Thermo Fisher Scientific) at 280 nm.

### 4.3. Toxicity Testing

The toxicity of proteinaceous extracts from *G. alba* proboscis and skin was assessed through a series of short-term ex vivo bioassays using gill tissue from the mussel *Mytilus* sp. as a biological model. After 48 h of acclimatization, thirty mussels were randomly assigned to the assays. Gills were harvested by separating the two valves, thus exposing demibranchs, and rinsing them with PBS (pH 7.4) to avoid desiccation. Tests were conducted immediately. For both organs (proboscis and skin) of *G. alba*, the protein fraction retained by 100 kDa microfilters was chosen and tested at a total protein concentration of 1 mg mL^−1^ (PBS was used as a biological control). The tests had six replicas, i.e., *n* = 6 per experimental condition (extracts and control). Tested valves were exposed to 1 mL of each test solution (including control, only exposed to cold PBS) for 10 min. After exposure, valves were immediately rinsed with PBS to avoid dehydration, and gill tissue was then carefully excised and divided into portions for subsequent analyses.

#### 4.3.1. Histopathology

Freshly harvested portions of gills exposed to protein extracts from *G. alba* skin and proboscis, plus controls, were fixated in Davidson’s solution (9–10% *v*/*v* formalin, 10% *v*/*v* glacial acetic acid and 30% ethanol prepared in Milli-Q-grade, >16 Ω.cm ultrapure water) for 24 h. Afterwards, samples were washed in ultrapure water (4 × 15 min), dehydrated through a progressive series of ethanol (70% for 5 min, 95% for 15 min and 100% for 35 min) and intermediately infiltrated with xylenes (20 min) before embedding in molten paraffin. Dehydration and embedding were performed under vacuum condition using a Shandon Pathcentre tissue processor (Thermo Fisher Scientific, Waltham, MA, USA). All samples were sectioned at a 5 μm thickness using a RM 2125 RTS rotary microtome (Leica Microsystems, Wetzlar, Germany). Then, sections were stained using hematoxylin and eosin, following Costa [41]. All slides were dehydrated, cleared with xylenes and mounted with DPX resin. Analyses were performed using a DM 2500 LED model microscope (Leica Microsystems).

#### 4.3.2. Comet Assay

Molecular damage in cells exposed to extracts was determined by analyzing DNA damage through an adaptation of the alkaline single-cell gel electrophoresis (comet) assay for solid tissue, after Martins and Costa [50], following Singh et al. [51]. Briefly, fresh gill samples were placed in 1.5 mL tubes with 200 μL of cold PBS (pH 7.4) gently with plyers and centrifuged at ≈1500× *g* at 4 °C for 2 min to precipitate debris. The supernatant containing live cells was collected in new tubes and mixed in 1% *m*/*v* molten (37–40 °C) low-melting-point agarose (LMPA) prepared in PBS. Afterwards, two 80 μL drops of the LMPA cell suspension were placed on slides precoated with 1.2% *m*/*v* of normal-melting-point agarose (dried for at least 48 h) and covered with a coverslip. After LMPA solidification (15 min, 4 °C), coverslips were removed and the slides were immersed in cold lysis buffer (0.45 M NaCl; 40 mM EDTA; 5 mM Tris; pH 10) for 1 h. Slides were then placed in cold electrophoresis buffer (0.1 mM EDTA, 0.3 M NaOH, pH 13) for 40 min to allow DNA unwinding and enhance the expression of alkali-labile sites. Electrophoresis was run at 25 V for 30 min at 4 °C. Then, the slides were neutralized in 0.2 M Tris-HCl, pH 7.5, and dried with methanol for archiving before analysis. Two slides (technical replicates) were prepared per sample. Rehydrated slides were stained with GreenSafe (Nzytech, Lisbon, Portugal) and analyzed with a DM 2500 LED model microscope equipped with an EL6000 model ultraviolet source and an MC 190 HD camera (all from Leica Microsystems). Micrographs of comet fields were obtained in grayscale mode at 400 × magnification. Scoring of micrographs was performed using the software CometScore 1.6 (TriTek Corporation, VA, USA), with 100 nucleoids analyzed per slide. The scoring metric obtained was % DNA in tail, which was considered a direct measure of DNA damage [52]. Additionally, the nucleoids were distributed into five classes of % DNA in tail (0–20, 20–40, 40–60, 60–80 and 80–100) for quality assessment and additional statistics.

#### 4.3.3. Caspase-3 Activity

As an indicator of programmed cellular death on mussel gills exposed to proteinaceous extracts from *G. alba* skin and proboscis, caspase-3 activity in apoptotic cells was determined using the Caspase-3 Colorimetric Assay Kit (Sigma-Aldrich). Briefly, cell lysates were obtained by adding 100 μL of lysis buffer (50 mM HEPES, 5 mM CHAPS and 5 mM DTT, pH 7.4) to approximately 30 mg of gill tissue, followed by maceration with a pestle. The samples were centrifuged at ≈14,000× *g* for 15 min at 4 °C. Supernatants were transferred to new tubes and stored at −80 °C until analyses. The procedure followed manufacturer instructions for 96-well plates. Plates were incubated for 90 min, and the optical density was measured using a Multiskan SkyHigh Microplate Spectrophotometer (Thermo Fisher Scientific). The activity of caspase-3 was estimated using a p-nitroaniline (pNA) standard curve. The final results were obtained by normalizing activity to total protein on cell lysates (μmol pNA min^−1^ mg^−1^).

### 4.4. Statistical Analysis

#### 4.4.1. Proteome Annotation and Search for Glycerotoxin (GLTx) Homologs

Using R 4.1.0+ [53] with the package UniprotR [54], peptides were annotated through homology matching, and corresponding gene ontology (GO) terms were retrieved (UniProtKB release 2023_01). As per recent updates to UniProtKB, peptides matching GLTx were assigned to the merged accession A0A1U9VX91. Then, BlastP (setting maximum *e*-value < 1 × 10^−5^) using the NCBI Blast+ suite 2.13.0 [47,48] was used to contrast peptides with at least 15 amino acids in length and matched to proboscis GLTx against the predicted coding regions from *G. alba* transcriptome described in Moutinho Cabral et al. [26] and deposited in the Gene Expression Omnibus (GEO) database (GSE196852). Further validation was obtained by contrasting peptides against all available GLTx entries (accessions A0A1U9VX91, A0A1U9VX95 and A0A1U9VX98, UniProtKB release 2022_01).

#### 4.4.2. Toxicity Testing

As previously performed, statistical analysis was computed with R. The nonparametric Dunn’s test was employed for multiple comparisons using the package ‘dunn.test’ [55], taking the protein extract from ‘organ’ as the independent (explanatory) variable with three factors (‘control’, ‘proboscis’ and ‘skin’). Dependent (response) variables were the ‘% DNA in Tail’ and normalized caspase-3 activity. The significance threshold (α) was set at 0.05 for all analyses.

## Figures and Tables

**Table 1:** Identification of glycerotoxins in crude protein extracts from *G. alba*’s proboscis and skin. The table shows the peptides’ putative protein matches and respective accessions, percentage of coverage with 95% confidence, indicated as % Cov (95), number of matched peptides and organ where the molecule was found.

Protein Match	Accession	% Cov (95)	Number of Matched Peptides	Organ
Glycerotoxin paralog 1 (*G. tridactyla*)	A0A1U9VX98	28.87–29.37	29	Proboscis
Glycerotoxin paralog 1 (*G. tridactyla*)	A0A1U9VX95	27.02–28.86	2–3	Proboscis
Glycerotoxin paralog 1 (*G. tridactyla*)	A0A1U9VX95	0.92	1	Skin

## Data Availability

Data are provided in the main text and Supplementary Information. Bulk data may be supplied upon request.

## Supplementary Materials

The following supporting information can be downloaded at: https://www.mdpi.com/article/10.3390/toxins15110655/s1, Table S1: Putative toxins that were identified in *G. alba*, with respective best-matched species in which the molecular mass has been described (kDa), Table S2: Identification of toxin- and venom-related proteins in *G. alba*, Figure S1: Venn diagram illustrating the distribution of the proteins identified in *Glycera alba*’s proboscis and skin, Figure S2: Top 5 GO terms of the proteins identified in common between the two organs, Figure S3: Top five GO terms among all identified proteins. Refs [56,57,58,59,60,61,62,63,64,65,66,67,68,69,70] are cited in the Supplementary Materials.
