# Supplementary material for: An Exploration of Novel Bioactives from the Venomous Marine Annelid Glycera alba
Source: Toxins (Basel). 2023 Nov 14;15(11):655. doi: 10.3390/toxins15110655 (PMC10674444; doi:10.3390/toxins15110655)
Supplement: Supplementary file 1 [file toxins-15-00655-s001.zip › toxins-2683735-supplementary.pdf]

Supplementary Materials: An exploration on novel bioactives from the venomous marine Polychaeta *Glycera alba*

Sónia Campos[1,2], Ana P. Rodrigo[1,2], Inês Moutinho cabral[1,2], Vera M. Mendes[3], Bruno Manadas[3], Mariaelena D'Ambrosio[1,2,*], Pedro M. Costa[1,2,*]

Table S1. Identified putative *G. alba* toxins, with respective species in which the molecular weight has been described (kDa). The potential toxins are classified into five functional categories, according to von Reumont et al. (2014).

Peptide	Molecular weight (kDa)	Category	Species	Reference
Actinoporin	20	Pore-forming	*G. dibranchiata*	von Reumont et al. [18]
SNTx-like toxin	150	Pore-forming	*S. horrida*	Nagai [56]
Sphingomyelinase	70-75	Pore-forming	Mammalian cells	Liscovitch [57]
Glycerotoxin	150	Neurotoxin	*G. tridactyla*	Richter et al. [22]
ShKT Domain	4	Neurotoxin	*S. helianthus*	Rangaraju et al. [58]
Cystatin	13	Protease inhibitors	All nucleated cells	Chew et al. [59]
Kunitz Domain	6	Protease inhibitors	*B. taurus*	Durani and Magliery [60]
Lipocalin	25	Protease inhibitors	*H. sapiens*	Singer et al. [61]
Serpin	35-50	Protease inhibitors	*T. molitor*	Jiang et al. [62]
CAP Domain	~15	CAP Domain	*S. cerevisiae*	Darwiche et al. [63]
C-type Lectin	15	Other enzymes	*T. nattereri*	Lopes-Ferreira et al. [64]
Chitinase	20-90	Other enzymes	Bacteria, fungi, plants, arthropods and humans	Hamid et al. [65]
Hyaluronidase	53	Other enzymes	*A. natalensis*	Barth et al. [66]
Phospholipase	16-18	Other enzymes	*A. cerana*	Shen et al. [67]
Peptidase S1	25-40	Other enzymes	Viperidae	Murayama et al. [68]
Peptidase S10	48	Other enzymes	*A. zychae*	Lee et al. [69]
Metalloproteinase M12	20-30	Other enzymes	*E. pennicornis*	Price et al. [70]

Table S2. Identification of toxin and venom-related proteins in Glycera alba. Peptidic sequences' protein match, with respective accession, number of matched peptides and percentage of coverage (95% confidence) - %Cov(95). P1 - Proboscis replicate 1; P2 - Proboscis replicate 2; S1 - Skin replicate 1; S2 - Skin replicate 2.

Accession	Peptide sP1	Peptide P2	Peptide sS1	Peptide sS2	%Cov(95) P1	%Cov(95) P2	%Cov(95) S1	%Cov(95) S2	Protein	Organism
A0A1U9VX98	29	29	NA	NA	28.87	29.37	NA	NA	Glycerotoxin paralog 1	*Glycera tridactyla*
A0A144KPJ6	9	9	4	7	2.52	3.45	1.49	1.61	Nonribosomal peptide synthetase TES	*Alternaria alternata*
Q01886	5	2	4	3	1.44	0.61	1.32	1.48	HC-toxin synthetase	*Cochliobolus carbonum*
Q12053	4	5	4	2	2.13	3.22	3.22	1.52	Norsolorinic acid synthase	*Aspergillus parasiticus*
A0A2G5IC96	4	NA	NA	NA	2.04	NA	NA	NA	Non-reducing polyketide synthase CTB1	*Cercospora beticola*
C9K7B5	4	6	3	1	1.56	1.63	1.26	0.32	AM-toxin synthetase AMT1	*Alternaria alternata*
C9K7I3	3	NA	NA	NA	5.46	NA	NA	NA	Aconitase AMT8-2	*Alternaria alternata*
Q01833	3	NA	1	1	2.12	NA	0.61	0.61	Complement C3 [Cleaved into: Complement C3 beta chain; Complement C3 alpha chain; C3a anaphylatoxin; Complement C3b alpha' chain; Complement C3f fragment]	*Naja naja*
V5Y0F7	3	2	2	1	1.92	1.36	1.96	0.55	Reducing polyketide synthase AFT16-1	*Alternaria alternata*
A0A084B9Z6	3	3	2	1	1.38	1.27	1.00	0.42	Non-reducing polyketide synthase SAT8	*Stachybotrys chartarum*

Accession	Peptide sP1	Peptide P2	Peptide sS1	Peptide sS2	%Cov(95) P1	%Cov(95) P2	%Cov(95) S1	%Cov(95) S2	Protein	Organism
D9N1A1	3	1	3	NA	1.06	0.28	1.63	NA	Highly reducing polyketide synthase ACTTS3	*Alternaria alternata*
A0A1U9VX95	2	3	NA	1	27.02	28.86	NA	0.92	Glycerotoxin paralog 1	*Glycera tridactyla*
P0DQE6	2	1	NA	1	18.44	11.35	NA	11.35	U-scoloptoxin(17)-Er3a	*Ethmostigmus rubripes*
P0DMY0	2	NA	NA	2	16.25	NA	NA	16.25	Kappa-actitoxin-Avd4f	*Anemonia viridis*
P07231	2	NA	1	NA	16.00	NA	8.00	NA	Conantokin-G	*Conus geographus*
A8S6B6	2	NA	NA	NA	10.92	NA	NA	NA	Cysteine-rich venom protein	*Austrelaps superbus*
A0A443HK11	2	3	NA	3	5.43	7.36	NA	7.71	FAD-binding monooxygenase VdtE	*Byssochlamys spectabilis*
B5U2W0	2	NA	NA	NA	5.00	NA	NA	NA	Venom serine protease Bi-VSP	*Bombus ignitus*
Q1PS45	2	NA	1	1	4.44	NA	3.95	1.97	Zinc metalloproteinase-disintegrin-like agkihagin	*Deinagkistrodon acutus*
A0A0D9MRV9	2	3	NA	1	4.17	4.92	NA	2.46	O-methylsterigmatocystin oxidoreductase	*Aspergillus flavus*
Q4WMK0	2	1	1	1	3.07	3.88	2.04	2.04	C6 finger domain transcription factor gliZ	*Neosartorya fumigata*
A0A3G9HRC2	2	1	NA	NA	2.09	2.72	NA	NA	Bifunctional cytochrome P450/NADPH--P450 reductase ALT2 [Includes: Cytochrome P450 monooxygenase; NADPH--cytochrome P450 reductase]	*Alternaria alternata*

Accession	Peptide sP1	Peptide P2	Peptide sS1	Peptide sS2	%Cov(95) P1	%Cov(95) P2	%Cov(95) S1	%Cov(95) S2	Protein	Organism
N4WHA7	2	1	NA	1	1.63	0.65	NA	0.37	Reducing polyketide synthase PKS2	*Cochliobolus heterostrophus*
Q2UVX4	2	3	NA	1	1.63	2.83	NA	0.72	Complement C3 [Cleaved into: Complement C3 beta chain; Complement C3 alpha chain; C3a anaphylatoxin; C3-beta-c; Acylation stimulating protein; Complement C3b alpha' chain; Complement C3c alpha' chain fragment 1; Complement C3dg fragment; Complement C3g fragment; Complement C3d fragment; Complement C3f fragment; Complement C3c alpha' chain fragment 2]	*Bos taurus*
Q8TGA2	2	NA	2	1	1.62	NA	1.32	0.90	Fatty acid synthase alpha subunit aflA [Includes: 3-oxoacyl-[acyl-carrier-protein] reductase; 3-oxoacyl-[acyl-carrier-protein] synthase]	*Aspergillus parasiticus*
A0A3G9H990	2	NA	1	2	1.56	NA	0.50	1.26	Highly reducing polyketide synthase ALT1	*Alternaria alternata*
Q4WMJ7	2	NA	NA	NA	1.41	NA	NA	NA	Nonribosomal peptide synthetase gliP	*Neosartorya fumigata*

Accession	Peptide sP1	Peptide P2	Peptide sS1	Peptide sS2	%Cov(95) P1	%Cov(95) P2	%Cov(95) S1	%Cov(95) S2	Protein	Organism
P12387	2	1	2	4	1.38	2.34	1.44	2.76	Complement C3 [Cleaved into: Complement C3 beta chain; C3-beta-c; Complement C3 alpha chain; C3a anaphylatoxin; Complement C3b alpha' chain; Complement C3c alpha' chain fragment 1; Complement C3dg fragment; Complement C3g fragment; Complement C3d fragment; Complement C3f fragment; Complement C3c alpha' chain fragment 2]	*Cavia porcellus*
I2C090	2	NA	NA	1	1.28	NA	NA	0.67	Ophiophagus venom factor [Cleaved into: OVF alpha chain; OVF gamma chain; OVF beta chain]	*Ophiophagus hannah*
A0A084API3	2	NA	1	3	1.05	NA	0.80	1.72	Highly reducing polyketide synthase SAT13	*Stachybotrys chartarum*
R4WH05	2	1	2	1	0.88	0.60	0.95	1.23	Highly reducing polyketide synthase ACRTS2	*Alternaria alternata*
P82421	1	1	NA	NA	45.83	45.83	NA	NA	U1-poneritoxin-Ng2a	*Neoponera goeldii*
Q45RU8	1	NA	NA	NA	25.00	NA	NA	NA	Conotoxin S5.1	*Conus striatus*
Q6PN83	1	NA	NA	NA	23.44	NA	NA	NA	Conotoxin mr5.3	*Conus marmoreus*

Accession	Peptides P1	Peptides P2	Peptides S1	Peptides S2	%Cov(95) P1	%Cov(95) P2	%Cov(95) S1	%Cov(95) S2	Protein	Organism
P69940	1	1	NA	NA	12.64	17.24	NA	NA	Potassium channel toxin TsTXK-beta	*Tityus serrulatus*
Q9BPA1	1	NA	NA	NA	12.09	NA	NA	NA	Conotoxin VnMKLT1-021	*Conus ventricosus*
Q9U659	1	1	NA	NA	11.69	11.69	NA	NA	Conotoxin PnMKLT1-0122	*Conus pennaceus*
P0DSK6	1	NA	NA	1	9.91	NA	NA	9.91	U-myrmeciitoxin(01)-Mg8b	*Myrmecia gulosa*
Q9YGI1	1	1	1	1	9.30	9.30	9.30	9.30	Probable weak neurotoxin NNAM3	*Naja atra*
Q9DEZ9	1	2	1	NA	7.47	11.20	7.47	NA	Venom nerve growth factor	*Crotalus durissus terrificus*
Q3HXY4	1	NA	NA	NA	7.11	NA	NA	NA	Venom nerve growth factor	*Pseudechis porphyriacus*
Q9BJV7	1	NA	NA	NA	7.00	NA	NA	NA	Omega-hexatoxin-Asp2b	*Atrax sp.*
P0DSK3	1	1	1	NA	6.67	6.67	6.67	NA	U-myrmeciitoxin(01)-Mg7a	*Myrmecia gulosa*
P0CAZ2	1	NA	NA	NA	6.20	NA	NA	NA	M-zodatoxin-Lt8i	*Lachesana tarabaevi*
P0DQA1	1	NA	NA	NA	6.17	NA	NA	NA	U-scoloptoxin(11)-Sm6a	*Scolopendra morsitans*
C0JB67	1	NA	NA	NA	5.82	NA	NA	NA	Dermonecrotic toxin SpeSicTox-betaIIA1	*Sicarius peruensis*
C0JB40	1	NA	NA	NA	5.11	NA	NA	NA	Dermonecrotic toxin LcsSicTox-betaIC1	*Loxosceles cf. spinulosa*
Q09GJ9	1	NA	NA	NA	5.02	NA	NA	NA	Cysteine-rich venom protein	*Philodryas olfersii*

Accession	Peptides P1	Peptide P2	Peptides S1	Peptides S2	%Cov(95) P1	%Cov(95) P2	%Cov(95) S1	%Cov(95) S2	Protein	Organism
A0A443HJZ3	1	NA	NA	NA	4.92	NA	NA	NA	Short-chain dehydrogenase/reductase VdtF	*Byssochlamys spectabilis*
B6H062	1	NA	1	NA	4.71	NA	4.71	NA	Short-chain dehydrogenase/reductase prx1	*Penicillium rubens*
Q8AY79	1	NA	1	NA	4.65	NA	3.10	NA	Beta-fibrinogenase stejnefibrase-2	*Trimeresurus stejnegeri*
P0CB14	1	NA	1	NA	4.08	NA	5.75	NA	Snake venom metalloproteinase kistomin	*Calloselasma rhodostoma*
P09807	1	1	NA	NA	4.02	4.02	NA	NA	Killer toxin subunit gamma	*Kluyveromyces lactis*
B5AJT3	1	NA	NA	NA	3.90	NA	NA	NA	Venom metalloproteinase 2	*Eulophus pennicornis*
A0A084API6	1	NA	NA	NA	3.80	NA	NA	NA	Trichothecene 15-O-acetyltransferase SAT16	*Stachybotrys chartarum*
Q4WTI4	1	NA	NA	NA	3.66	NA	NA	NA	Transcription factor rglT	*Neosartorya fumigata*
A0A1L4BJ98	1	NA	NA	NA	3.40	NA	NA	NA	Dermonecrotic toxin Hl-PLD1	*Hemiscorpius lepturus*
Q7M4I3	1	NA	NA	1	3.29	NA	NA	3.29	Venom protease	*Bombus pensylvanicus*
P0DSI2	1	1	NA	NA	2.90	2.90	NA	NA	Phospholipase A1	*Dinoponera quadriceps*
A0A3G9HPE6	1	NA	NA	NA	2.87	NA	NA	NA	Dehydrogenase ALT3	*Alternaria alternata*
Q8I913	1	NA	NA	NA	2.57	NA	NA	NA	Dermonecrotic toxin LlSicTox-alphaIII3i	*Loxosceles laeta*

Accession	Peptides P1	Peptide P2	Peptides S1	Peptides S2	%Cov(95) P1	%Cov(95) P2	%Cov(95) S1	%Cov(95) S2	Protein	Organism
A0ST43	1	NA	NA	NA	2.40	NA	NA	NA	FAD-dependent monooxygenase CTB5	*Cercospora nicotianae*
B7W114	1	1	1	2	2.33	2.33	2.33	4.86	Cephalotoxin-like protein	*Acropora millepora*
Q8AWX7	1	NA	NA	NA	2.25	NA	NA	NA	Zinc metalloproteinase-disintegrin agkistin	*Gloydius halys*
P98093	1	3	2	1	2.13	4.15	1.59	0.67	Complement C3 [Cleaved into: Complement C3 beta chain; Complement C3 alpha chain; C3a anaphylatoxin; Complement C3b alpha' chain; Complement C3c alpha' chain fragment 1; Complement C3dg fragment; Complement C3g fragment; Complement C3d fragment; Complement C3f fragment; Complement C3c alpha' chain fragment 2]	*Oncorhynchus mykiss*
A0A084B9Z8	1	2	NA	NA	2.12	1.19	NA	NA	Ankyrin repeat domain-containing protein SAT10	*Stachybotrys chartarum*
A0A3G9H9H8	1	1	1	1	1.99	1.99	1.99	1.99	Nonribosomal peptide synthetase ALT12	*Alternaria alternata*
Q98989	1	1	NA	1	1.71	1.71	NA	1.71	Stonustoxin subunit alpha	*Synanceia horrida*
A2R6H0	1	NA	1	NA	1.60	NA	0.59	NA	Nonribosomal peptide synthetase otaB	*Aspergillus niger*
Q2UXQ5	1	NA	NA	NA	1.47	NA	NA	NA	Zinc metalloproteinase-disintegrin-like EoVMP2	*Echis ocellatus*

Accession	Peptide sP1	Peptide P2	Peptide sS1	Peptide sS2	%Cov(95) P1	%Cov(95) P2	%Cov(95) S1	%Cov(95) S2	Protein	Organism
Q23647	1	NA	NA	NA	1.41	NA	NA	NA	Hypersensitive to pore-forming toxin protein 40	*Caenorhabditis elegans*
Q2I0M6	1	2	NA	NA	1.38	2.87	NA	NA	Dual O-methyltransferase/FAD-dependent monooxygenase CTB3 [Includes: O-methyltransferase; FAD-dependent monooxygenase]	*Cercospora nicotianae*
Q0ZZJ6	1	4	NA	NA	1.15	2.48	NA	NA	*A.superbus* venom factor 1 [Cleaved into: AVF-1 alpha chain; AVF-1 gamma chain; AVF-1 beta chain]	*Austrelaps superbus*
A0A2D0TC04	1	NA	NA	NA	0.96	NA	NA	NA	Venom phosphodiesterase	*Naja atra*
Q8TGA1	1	2	NA	NA	0.95	1.06	NA	NA	Fatty acid synthase beta subunit aflB [Includes: 3-hydroxyacyl-[acyl-carrier-protein] dehydratase; Enoyl-[acyl-carrier-protein] reductase [NADH]; [Acyl-carrier-protein] acetyltransferase; [Acyl-carrier-protein] malonyltransferase; S-acyl fatty acid synthase thioesterase]	*Aspergillus parasiticus*
A0A125SUR3	1	2	NA	2	0.80	2.39	NA	1.13	Nonribosomal peptide synthetase ACTTS4	*Alternaria alternata*

**Accession**	**Peptides P1**	**Peptides P2**	**Peptides S1**	**Peptides S2**	**%Cov(95) P1**	**%Cov(95) P2**	**%Cov(95) S1**	**%Cov(95) S2**	**Protein**	**Organism**
Q58L91	1	NA	NA	NA	0.75	NA	NA	NA	Venom prothrombin activator oscutarin-C non-catalytic subunit [Cleaved into: Oscutarin-C non-catalytic subunit heavy chain; Oscutarin-C non-catalytic subunit light chain]	*Oxyuranus scutellatus*
P98094	1	4	1	NA	0.74	2.96	0.74	NA	Complement C3 [Cleaved into: Complement C3 beta chain; Complement C3 alpha chain; C3a anaphylatoxin; Complement C3 gamma chain]	*Eptatretus burgeri*
A2R6H1	1	1	1	2	0.71	1.88	1.18	1.10	Highly reducing polyketide synthase otaA	*Aspergillus niger*
P08649	1	NA	NA	1	0.69	NA	NA	0.52	Complement C4 [Cleaved into: Complement C4 beta chain; Complement C4 alpha chain; C4a anaphylatoxin; Complement C4 gamma chain]	*Rattus norvegicus*
A0A1R3RGK1	1	2	NA	NA	0.59	0.96	NA	NA	Nonribosomal peptide synthetase otaB	*Aspergillus carbonarius*
A0A1R3RGK0	1	6	NA	1	0.39	3.66	NA	0.51	Highly reducing polyketide synthase otaA	*Aspergillus carbonarius*
A0A443HK81	1	1	1	2	0.31	0.31	0.31	0.70	Non-reducing polyketide synthase VdtA	*Byssochlamys spectabilis*
N4WHE3	1	1	1	2	0.28	1.35	0.32	1.27	Reducing polyketide synthase PKS1	*Cochliobolus heterostrophus*

Accession	Peptides P1	Peptide P2	Peptides S1	Peptides S2	%Cov(95) P1	%Cov(95) P2	%Cov(95) S1	%Cov(95) S2	Protein	Organism
B2DCR8	NA	3	1	NA	NA	3.14	0.86	NA	SE-cephalotoxin	*Sepia esculenta*
Q00685	NA	3	2	1	NA	2.09	2.33	0.72	Complement C3 [Cleaved into: Complement C3 beta chain; Complement C3 alpha chain; C3a anaphylatoxin; Complement C3 gamma chain]	*Lethenteron camtschaticum*
Q6DQW3	NA	3	5	4	NA	1.59	2.69	2.23	Non-reducing polyketide synthase CTB1	*Cercospora nicotianae*
P01026	NA	2	NA	NA	NA	1.68	NA	NA	Complement C3 [Cleaved into: Complement C3 beta chain; C3-beta-c; Complement C3 alpha chain; C3a anaphylatoxin; Acylation stimulating protein; Complement C3b alpha' chain; Complement C3c alpha' chain fragment 1; Complement C3dg fragment; Complement C3g fragment; Complement C3d fragment; Complement C3f fragment; Complement C3c alpha' chain fragment 2]	*Rattus norvegicus*
J3S836	NA	2	3	NA	NA	1.03	2.18	NA	Venom factor [Cleaved into: VF alpha chain; VF gamma chain; VF beta chain]	*Crotalus adamanteus*
A1X8D1	NA	1	1	1	NA	44.74	44.74	44.74	Alpha-conotoxin-like Qc1.6	*Conus quercinus*

Accession	Peptides P1	Peptide P2	Peptides S1	Peptides S2	%Cov(95) P1	%Cov(95) P2	%Cov(95) S1	%Cov(95) S2	Protein	Organism
Q9BPB2	NA	1	NA	NA	NA	17.81	NA	NA	Conotoxin Asp7/Gla(3)-TxVI	*Conus textile*
A1X8B6	NA	1	NA	NA	NA	16.18	NA	NA	Alpha-conotoxin Lp1.4	*Conus leopardus*
Q75WH3	NA	1	NA	NA	NA	14.29	NA	NA	U11-hexatoxin-Mg1a	*Macrothele gigas*
D2Y2L0	NA	1	NA	NA	NA	13.10	NA	NA	U4-theraphotoxin-Hhn1a	*Cyriopagopus hainanus*
P0DPU9	NA	1	NA	NA	NA	11.94	NA	NA	U-myrmeciitoxin(01)-Mg4a	*Myrmecia gulosa*
C0HJV4	NA	1	NA	NA	NA	11.43	NA	NA	Cytoinsectotoxin-2c	*Lachesana tarabaevi*
Q0GY44	NA	1	NA	NA	NA	10.34	NA	NA	Potassium channel toxin Tdi-beta-KTx	*Tityus discrepans*
A0A0D4WTV1	NA	1	NA	NA	NA	8.65	NA	NA	Dermonecrotic toxin LarSicTox-betaID1	*Loxosceles arizonica*
Q1ELU7	NA	1	NA	NA	NA	8.62	NA	NA	M-zodatoxin-Lt6a/c [Cleaved into: M-zodatoxin-Lt6c; M-zodatoxin-Lt6a]	*Lachesana tarabaevi*
P35775	NA	1	NA	NA	NA	7.97	NA	NA	Venom allergen 2	*Solenopsis invicta*
A0A6B9L6A5	NA	1	NA	1	NA	7.08	NA	3.75	Venom hemolysin-like protein 1	*Platymeris rhadamanthus*
K9USW8	NA	1	1	NA	NA	5.00	5.00	NA	Dermonecrotic toxin LgSicTox-alphaIC1 [Cleaved into: U1-sicaritoxin-Lg1a]	*Loxosceles gaucho*
C0JAS3	NA	1	NA	NA	NA	4.43	NA	NA	Dermonecrotic toxin LhSicTox-alphaIA2bv	*Loxosceles hirsuta*

Accession	Peptide sP1	Peptide P2	Peptide sS1	Peptide sS2	%Cov(95) P1	%Cov(95) P2	%Cov(95) S1	%Cov(95) S2	Protein	Organism
C0JB28	NA	1	NA	NA	NA	4.35	NA	NA	Dermonecrotic toxin LlSicTox-alphaIV2iv	*Loxosceles laeta*
A0A125SUR4	NA	1	NA	NA	NA	4.20	NA	NA	Nonribosomal peptide synthetase ACTTS1	*Alternaria alternata*
B6H060	NA	1	NA	NA	NA	3.02	NA	NA	Short-chain dehydrogenase/reductase prx6	*Penicillium rubens*
C0JB13	NA	1	NA	NA	NA	2.88	NA	NA	Dermonecrotic toxin LlSicTox-alphaIII3iii	*Loxosceles laeta*
Q5BLY5	NA	1	NA	NA	NA	2.83	NA	NA	Venom acid phosphatase Acph-1	*Apis mellifera*
E5AJX2	NA	1	NA	NA	NA	2.72	NA	NA	Snake venom serine protease nikobin	*Vipera berus nikolskii*
P30431	NA	1	3	NA	NA	2.45	5.60	NA	Zinc metalloproteinase-disintegrin-like jararhagin [Cleaved into: Disintegrin-like jararhagin-C]	*Bothrops jararaca*
C9K7B8	NA	1	1	NA	NA	2.40	2.40	NA	Isopropyl malate synthase AMT7	*Alternaria alternata*
Q6UEG8	NA	1	1	NA	NA	2.25	2.25	NA	Aflatoxin biosynthesis regulatory protein	*Aspergillus parasiticus*
D8VNS0	NA	1	NA	NA	NA	1.95	NA	NA	Zinc metalloproteinase-disintegrin-like	*Cerberus rynchops*
P58912	NA	1	NA	NA	NA	1.84	NA	NA	DELTA-alicitoxin-Pse2b	*Phyllodiscus semoni*
J3SBP3	NA	1	NA	NA	NA	1.24	NA	NA	Venom phosphodiesterase 2	*Crotalus adamanteus*

Accession	Peptides P1	Peptide P2	Peptides S1	Peptides S2	%Cov(95) P1	%Cov(95) P2	%Cov(95) S1	%Cov(95) S2	Protein	Organism
P0DPU3	NA	1	NA	NA	NA	1.21	NA	NA	Scoloptoxin SSD14 [Cleaved into: SLPTX-SSD14 subunit alpha; SLPTX-SSD14 subunit beta]	*Scolopendra dehaani*
A0A443HK66	NA	1	1	NA	NA	0.78	0.78	NA	Highly reducing polyketide synthase VdtX	*Byssochlamys spectabilis*
Q50LG3	NA	1	3	NA	NA	0.77	1.43	NA	Highly reducing polyketide synthase AFT9-1	*Alternaria alternata*
D7PHZ2	NA	1	1	3	NA	0.71	0.71	1.48	Non-reducing polyketide synthase vrtA	*Penicillium aethiopicum*
P01029	NA	1	NA	NA	NA	0.63	NA	NA	Complement C4-B [Cleaved into: Complement C4 beta chain; Complement C4 alpha chain; C4a anaphylatoxin; Complement C4 gamma chain]	*Mus musculus*
A0A1R3RGJ2	NA	NA	3	NA	NA	NA	6.83	NA	Flavin-dependent halogenase otaD	*Aspergillus carbonarius*

Accession	Peptides P1	Peptide P2	Peptides S1	Peptides S2	%Cov(95) P1	%Cov(95) P2	%Cov(95) S1	%Cov(95) S2	Protein	Organism
P01027	NA	NA	3	1	NA	NA	2.77	0.72	Complement C3 [Cleaved into: Complement C3 beta chain; C3-beta-c; Complement C3 alpha chain; C3a anaphylatoxin; Acylation stimulating protein; Complement C3b alpha' chain; Complement C3c alpha' chain fragment 1; Complement C3dg fragment; Complement C3g fragment; Complement C3d fragment; Complement C3f fragment; Complement C3c alpha' chain fragment 2]	*Mus musculus*
Q6PTD5	NA	NA	2	1	NA	NA	29.41	22.06	Alpha-conotoxin Lp1.1	*Conus leopardus*
E2JFG2	NA	NA	2	NA	NA	NA	4.52	NA	Dual function macrocyclase-peptidase POPB	*Amanita bisporigera*
C5NN18	NA	NA	2	NA	NA	NA	3.63	NA	Acyl-CoA synthetase ACTT5	*Alternaria alternata*
C9K7C0	NA	NA	2	NA	NA	NA	3.53	NA	O-methyltransferase AMT9	*Alternaria alternata*
P82426	NA	NA	1	NA	NA	NA	50.00	NA	M-poneritoxin-Ng1d	*Neoponera goeldii*
H9N3R7	NA	NA	1	NA	NA	NA	48.39	NA	Alpha-conotoxin Li1.12	*Conus lividus*
P0DSL8	NA	NA	1	1	NA	NA	45.45	45.45	U1-pseudomyrmecitoxin-Pt1 subunit LS2	*Pseudomyrmex triplarinus*

Accession	Peptides P1	Peptide P2	Peptides S1	Peptides S2	%Cov(95) P1	%Cov(95) P2	%Cov(95) S1	%Cov(95) S2	Protein	Organism
D2Y170	NA	NA	1	1	NA	NA	22.37	22.37	Conotoxin Cal5a L2 [Cleaved into: Conotoxin Cal5b L2; Conotoxin Cal5.1]	*Californiconus californicus*
P0DPY2	NA	NA	1	NA	NA	NA	15.63	NA	U-scoloptoxin(06)-Sm1a	*Scolopendra morsitans*
P0DPV1	NA	NA	1	NA	NA	NA	11.34	NA	U-scoloptoxin(10)-Ssd2a	*Scolopendra dehaani*
P0DQP2	NA	NA	1	NA	NA	NA	9.52	NA	Venom protein SynTx	*Dendroaspis jamesoni jamesoni*
D9U2A1	NA	NA	1	NA	NA	NA	9.30	NA	Neurotoxin LmNaTx17	*Lychas mucronatus*
P82114	NA	NA	1	1	NA	NA	8.26	8.26	Basic phospholipase A2 homolog MjTX-I	*Bothrops moojeni*
D6C4I6	NA	NA	1	NA	NA	NA	8.07	NA	Conotoxin Cl14.12	*Californiconus californicus*
Q4LCT2	NA	NA	1	NA	NA	NA	7.41	NA	Toxin-like peptide AaF1CA5	*Androctonus australis*
Q3HXY3	NA	NA	1	2	NA	NA	7.02	7.02	Venom nerve growth factor 1	*Pseudechis australis*
Q9BJV8	NA	NA	1	1	NA	NA	7.00	7.00	Omega-hexatoxin-Asp2a	*Atrax sp.*
P0DPZ8	NA	NA	1	NA	NA	NA	5.86	NA	U-scoloptoxin(11)-Sm3a	*Scolopendra morsitans*
D2Y255	NA	NA	1	NA	NA	NA	5.31	NA	U11-theraphotoxin-Hhn1a	*Cyriopagopus hainanus*
W6Q4Q9	NA	NA	1	NA	NA	NA	5.26	NA	Aristolochene synthase	*Penicillium roqueforti*

Accession	Peptide sP1	Peptide P2	Peptide sS1	Peptide sS2	%Cov(95) P1	%Cov(95) P2	%Cov(95) S1	%Cov(95) S2	Protein	Organism
C9K1N0	NA	NA	1	NA	NA	NA	5.09	NA	Abhydrolase domain-containing protein ACTT2-3	*Alternaria alternata*
Q8QG86	NA	NA	1	1	NA	NA	3.89	3.89	Snake venom serine protease BITS01A	*Bothrops insularis*
A0A2G5ICB4	NA	NA	1	NA	NA	NA	3.02	NA	Cercosporin biosynthesis regulatory protein CTB8	*Cercospora beticola*
Q4WMJ9	NA	NA	1	NA	NA	NA	2.99	NA	Probable aminotransferase gliI	*Neosartorya fumigata*
D7PI00	NA	NA	1	NA	NA	NA	2.10	NA	Transcription factor vrtR1	*Penicillium aethiopicum*
C9K7C2	NA	NA	1	NA	NA	NA	2.08	NA	Transcription activator AMTR1	*Alternaria alternata*
A0A2G5IC53	NA	NA	1	NA	NA	NA	1.61	NA	Dual O-methyltransferase/FAD-dependent monooxygenase CTB3 [Includes: O-methyltransferase; FAD-dependent monooxygenase]	*Cercospora beticola*
Q50LG1	NA	NA	1	NA	NA	NA	1.37	NA	Cytochrome P450 monooxygenase AFT11-1	*Alternaria alternata*
K4RNX9	NA	NA	NA	1	NA	NA	NA	26.83	Alpha-conotoxin TxIB	*Conus textile*
P0CJ19	NA	NA	NA	1	NA	NA	NA	22.03	Venom protein 27.7	*Lychas mucronatus*
A3F7X2	NA	NA	NA	1	NA	NA	NA	11.21	U3-theraphotoxin-Lsp1a	*Lasiodora sp.*

Accession	Peptides P1	Peptide P2	Peptides S1	Peptides S2	%Cov(95) P1	%Cov(95) P2	%Cov(95) S1	%Cov(95) S2	Protein	Organism
A8YPR6	NA	NA	NA	1	NA	NA	NA	7.47	Snake venom metalloprotease inhibitor 02D01 [Cleaved into: Tripeptide pEKW 1; Tripeptide pEKW 2; Tripeptide pEKW 3; Tripeptide pEKW 4; Tripeptide pEKW 5; Tripeptide pEKW 6; Tripeptide pEKW 7; Tripeptide pEKW 8; Tripeptide pEKW 9; Tripeptide pEKW 10; Tripeptide pEKW 11; Poly-His-poly-Gly peptide 4; Poly-His-poly-Gly peptide 3; Poly-His-poly-Gly peptide 2; Poly-His-poly-Gly peptide 1; C-type natriuretic peptide]	*Echis ocellatus*
Q1W694	NA	NA	NA	1	NA	NA	NA	6.89	Dermonecrotic toxin LiSicTox-betaID1	*Loxosceles intermedia*
Q2XXP4	NA	NA	NA	1	NA	NA	NA	4.66	Cysteine-rich venom protein TRI1	*Trimorphodon biscutatus*
C0JAS6	NA	NA	NA	1	NA	NA	NA	4.40	Dermonecrotic toxin LspaSicTox-alphaIA2i	*Loxosceles spadicea*
D7UPN2	NA	NA	NA	1	NA	NA	NA	4.37	Trans-enoyl reductase ACTTS2	*Alternaria alternata*

Accession	Peptides P1	Peptides P2	Peptides S1	Peptides S2	%Cov(95) P1	%Cov(95) P2	%Cov(95) S1	%Cov(95) S2	Protein	Organism
Q1ELU5	NA	NA	NA	1	NA	NA	NA	4.35	M-zodatoxin-Lt4a [Cleaved into: Repetitive polypeptide element type 1a; Repetitive polypeptide element type 1b; M-zodatoxin-Lt4a peptide]	*Lachesana tarabaevi*
A2R6G7	NA	NA	NA	1	NA	NA	NA	3.15	Flavin-dependent halogenase otaD	*Aspergillus niger*
Q63530	NA	NA	NA	1	NA	NA	NA	2.87	Phosphotriesterase-related protein	*Rattus norvegicus*
A0A2P1BRP3	NA	NA	NA	1	NA	NA	NA	2.14	Cytolytic toxin-beta	*Scorpaena plumieri*
C9K7F3	NA	NA	NA	1	NA	NA	NA	2.09	AM-toxin biosynthesis protein 12-2	*Alternaria alternata*
P0C7B0	NA	NA	NA	1	NA	NA	NA	1.48	Zinc metalloproteinase-disintegrin-like brevilysin H6 [Cleaved into: p45K; Disintegrin-like p29K]	*Gloydius brevicaudus*

Accession	Peptides P1	Peptides P2	Peptides S1	Peptides S2	%Cov(95) P1	%Cov(95) P2	%Cov(95) S1	%Cov(95) S2	Protein	Organism
P01025	NA	NA	NA	1	NA	NA	NA	0.48	Complement C3 [Cleaved into: Complement C3 beta chain; C3-beta-c; Complement C3 alpha chain; C3a anaphylatoxin; Acylation stimulating protein; Complement C3b alpha' chain; Complement C3c alpha' chain fragment 1; Complement C3dg fragment; Complement C3g fragment; Complement C3d fragment; Complement C3f fragment; Complement C3c alpha' chain fragment 2]	*Sus scrofa*

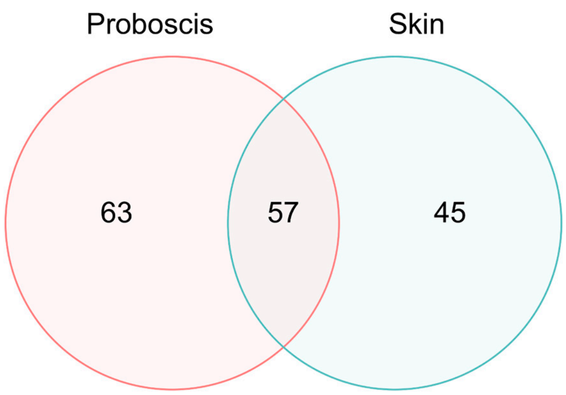

Figure S1. Veen Diagram illustrating the distribution of the proteins identified in *Glycera alba*'s proboscis and skin.

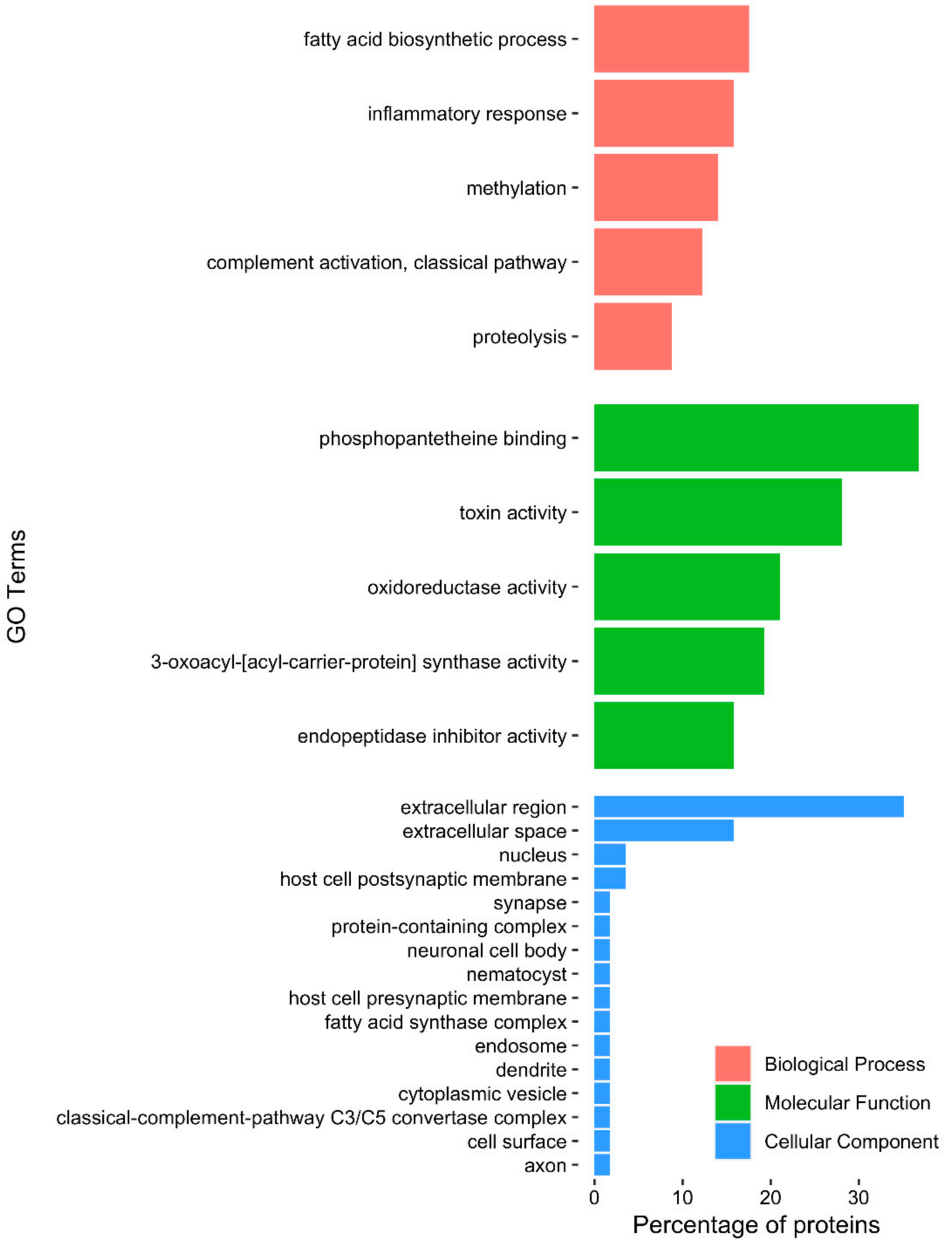

Figure S2. Top5 GO Terms of the proteins identified in common between the two organs. The horizontal bar chart shows the percentage of the proteins identified in common between *Glycera alba*'s proboscis and skin. related to each GO Term. The same protein can bear multiple GO Terms.

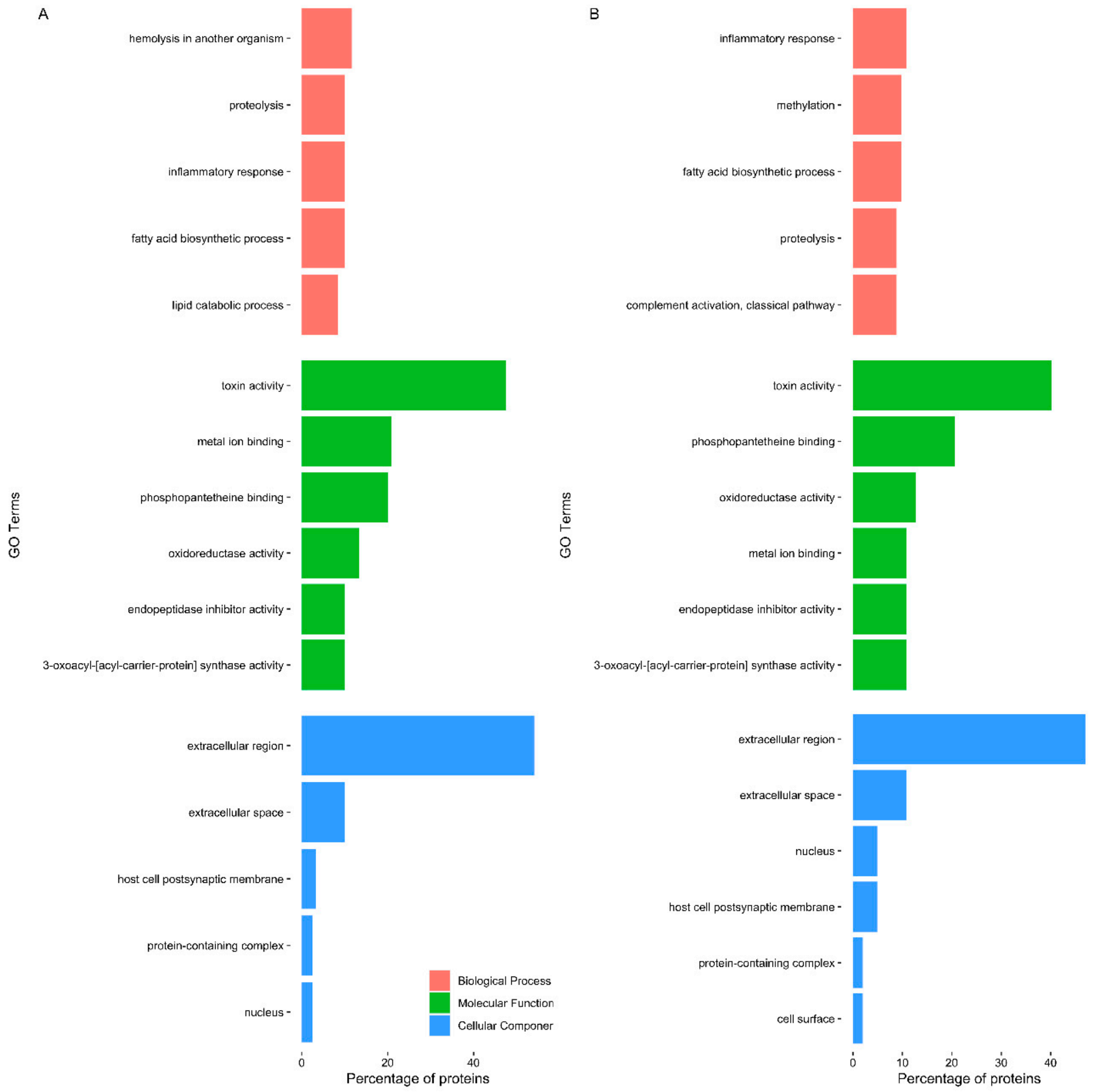

Figure S3. Top5 GO Terms of all proteins identified. The horizontal bar chart shows the percentage of all proteins identified. A) in *Glycera alba*'s proboscis. B) in *Glycera alba*'s skin. related to each GO Term. The same protein can bear multiple GO Terms.

References

18. von Reumont, B.M.; Campbell, L.I.; Richter, S.; Hering, L.; Sykes, D.; Hetmank, J.; Jenner, R.A.; Bleidorn, C. A polychaete's powerful punch: Venom gland transcriptomics of *Glycera* reveals a complex cocktail of toxin homologs. Genome Biol. Evol. 2014, 6, 2406–2423, doi: 10.1093/gbe/evu190.
22. Richter, S.; Helm, C.; Meunier, F.A.; Hering, L.; Campbell, L.I.; Drukewitz, S.H.; Undheim, E.A.B.; Jenner, R.A.; Schiavo, G.; Bleidorn, C. Comparative analyses of glycerotoxin expression unveil a novel structural organization
56. Nagai, H. Marine protein toxins. In Handbook of Marine Natural Products; Fattorusso, E., Gerwick, W., Taglialatela-Scafati, O.; Springer: Dordrecht, 2012; 1389-1420, doi:10.1007/978-90-481-3834-0_29.
57. Liscovitch, M. Receptor-regulated phospholipases. Encyclopedia of Endocrine Diseases. 2004, 4, 167-173.
58. Rangaraju, S.; Khoo, K.K.; Feng, Z.P.; Crossley, G.; Nugent, D.; Khaytin, I.; Chi, V.; Pham, C.; Calabresi, P.; Pennington, M.W.; et al. Potassium channel modulation by a toxin domain in matrix metalloprotease 23. J Biol Chem. 2010, 285, 9124-9136, doi:10.1074/jbc.M109.071266.
59. Chew, J.S.C.; Saleem, M.; Florkowski C.M.; George, P.M. Cystatin C–A Paradigm of evidence based laboratory medicine. Clin Bio-chem Rev. 2008, 29, 47-62, PMID 18787643.
60. Durani, V.; Magliery, T.J. Protein engineering and stabilization from sequence statistics: variation and covariation analysis. Meth. Enzymol. 2013, 523, 237-256, doi:10.-1016/B978-0-12-394292-0.00011-4.
61. Singer, E.; Markó, L.; Paragas, N.; Barasch, J.; Dragun, D.; Müller, D.N.; Budde, K.; Schmidt-Ott, K.M. Neutrophil gelati-nase-associated lipocalin: pathophysiology and clinical applications. Acta. Physiol. 2013, 207, 663-672, doi:10.1111/apha.12054.
62. Jiang, R.; Zhang, B.; Kurokawa, K.; So, Y.; Kim, E.; Hwang, H.O.; Lee, J.; Shiratsuchi, A.; Zhang, J.; Nakanishi, Y. et al. 93-kDa Twin-domain serine protease inhibitor (serpin) has a regulatory function on the beetle toll proteolytic signaling cascade. J. Biol. Chem. 2011, 286, 35087-35095, doi:10.1074/jbc.M111.277343.
63. Darwiche, R.; Kelleher, A.; Hudspeth E.M; Schneiter, R.; Asojo, O.A. Structural and functional characterization of the CAP domain of pathogen-related yeast 1 (Pry1) protein. Sci. Rep. 2006, 6, 28838, doi:10.1038/srep28838.
64. Lopes-Ferreira, M.; Magalhães, G.S.; Fernandez J.H.; Junqueira-de-Azevedo, I.d.L.; Le Ho, P.; Lima, C.; Valente, R.H.; Moura-da-Silva, A.M. Structural and biological characterization of Nattectin, a new C-type lectin from the venomous fish *Thalassophryne nattereri*. Biochimie. 2011, 93, 971-980, doi:10.1016/j.biochi.2011.03.001.
65. Hamid, R. et al., 2013. Chitinases: An update. J Pharm Bioallied Sci. 5, 21-29, doi: 10.4103/0975-7406.106559.
66. Barth, T.; Coelho Mandacaru, S.; Charneau, S.; Valle de Souza, M.; Ornelas Ricart, C.A.; Ferreira Noronha, E.; Araújo Souza, A.; de Freitas, S.M.; Roespstorff, P.; Fontes, W. et al. Biochemical and structural characterization of a protein complex con-taining a hyaluronidase and a CRISP-like protein isolated from the venom of the spider *Acanthoscurria natalensis*. J Proteomics. 2019, 192, 102-113, doi:10.1016/j.jprot.2018.08.012.
67. Shen, L.R.; Ding, M.H.; Zhang, L.W.; Zhang, W.G.; Liu, L.; Li, D. Expression of a bee venom phospholipase A2 from *Apis cerana cerana* in the baculovirus-insect cell. J Zhejiang Univ Sci B. 2010, 11, 342-349. doi:10.1631/jzus.B0900254.
68. Murayama, N.; Saguchi, K.; Mentele, R.; Assakura, M.T.; Ohi, H.; Fujita, Y.; Camargo, A.C.; Higuchi, S.; Serrano, S.M. The unusual high molecular mass of Bothrops protease A, a trypsin-like serine peptidase from the venom of *Bothrops jararaca*, is due to its high carbohydrate content. Biochim Biophys Acta Proteins Proteom. 2003, 1652, 1-6, doi:10.1016/j.bbapap.2003.08.001.
69. Lee, B.R.; Takeuchi, M.; Kobayashi, Y. Purification and characterization of serine carboxypeptidases from *Absidia zychae*. Biosci. Biotech. Biochem. 1993, 57, 618-622. doi:10.1271/bbb-.57.618.

70. Price, D.R.G.; Bell, H.A.; Hinchliffe, G.; Fitches, E.; Weaver, R.; Gatehouse, J.A. A venom metalloproteinase from the parasitic wasp *Eulophus pennicornis* is toxic towards its host, tomato moth (*Lacanobia oleracae*). Insect Mol Biol. 2009, 18, 195-202. doi:10.1111/j.1365-2583.2009.00864.x.of the bloodworm venom system. BMC Evol. Biol. **2017**, 17, 1–19, doi: 10.1186/s12862-017-0904-4
